# Long-term effects of stimulant exposure on cerebral blood flow response to methylphenidate and behavior in attention-deficit hyperactivity disorder

**DOI:** 10.1007/s11682-017-9707-x

**Published:** 2017-03-20

**Authors:** Anouk Schrantee, C. Bouziane, E. E. Bron, S. Klein, M. A. Bottelier, J. J. S. Kooij, S. A. R. B. Rombouts, L. Reneman

**Affiliations:** 1 Department of Radiology, Academic Medical Center, University of Amsterdam, P.O. Box 22660, 1105 AZ Amsterdam, the Netherlands; 2 Biomedical Imaging Group Rotterdam, Departments of Medical Informatics and Radiology, Erasmus MC, P.O. Box 2040, 3000 CA Rotterdam, The Netherlands; 3 Department of Child- and Adolescent Psychiatry, Triversum, Kees Boekestraat 5, 1817 EZ Alkmaar, The Netherlands; 4 Expertise Center Adult ADHD, PsyQ, Psycho-Medical Programs, Carel Reinierszkade 197, 2593 HR The Hague, The Netherlands; 5 Institute of Psychology, Leiden University, P.O. Box 9555, 2300 RB Leiden, The Netherlands; 6 Department of Radiology, LUMC, P.O. Box 9600, 2300 RC, Leiden, The Netherlands

**Keywords:** ADHD, Methylphenidate, Stimulants, phMRI, Age, CBF

## Abstract

Stimulant prescription rates for attention deficit hyperactivity disorder (ADHD) are increasing, even though potential long-term effects on the developing brain have not been well-studied. A previous randomized clinical trial showed short-term age-dependent effects of stimulants on the DA system. We here assessed the long-term modifying effects of age-of-first-stimulant treatment on the human brain and behavior. 81 male adult ADHD patients were stratified into three groups: 1) *early stimulant treatment (EST;* <16 years of age) 2) *late stimulant treatment (LST*: ≥23 years of age) and 3) *stimulant treatment naive (STN;* no history of stimulant treatment). We used pharmacological magnetic resonance imaging (phMRI) to assess the cerebral blood flow (CBF) response to an oral methylphenidate challenge (MPH, 0.5 mg/kg), as an indirect measure of dopamine function in fronto-striatal areas. In addition, mood and anxiety scores, and recreational drug use were assessed. Baseline ACC CBF was lower in the *EST* than the *STN* group (*p* = 0.03), although CBF response to MPH was similar between the three groups (*p* = 0.23). ADHD symptom severity was higher in the *STN* group compared to the other groups (*p* < 0.01). In addition, the *EST* group reported more depressive symptoms (*p* = 0.04), but not anxiety (*p* = 0.26), and less recreational drug use (*p* = 0.04). In line with extensive pre-clinical data, our data suggest that early, but not late, stimulant treatment long-lastingly affects the human brain and behavior, possibly indicating fundamental changes in the dopamine system.

## Introduction

Attention-deficit hyperactivity disorder (ADHD) is one of the most common psychiatric disorders diagnosed in children and adolescents (Thomas et al. 2015), and also has a high prevalence in adults (approximately 2.5%) (Simon et al. 2009). The most prescribed treatment for ADHD is stimulant medication, such as methylphenidate (MPH) and dexamphetamine (dAMPH). Stimulants act upon the dopamine (DA) neurotransmitter system by increasing extracellular DA, and have been shown to be very effective in reducing behavioral symptoms in ADHD (van de Loo-Neus et al. 2011). However, as prescription rates of stimulants are rising (McCarthy et al. 2012), concern about potential long-term consequences of stimulants on the developing DA system is increasingly being voiced by a number of entities. These include parents of patients, healthcare professionals, the Food and Drug Administration (FDA) and National Institutes of Health (NIH).

Prospective studies are the ideal study design to investigate the long-term effects of stimulants on the development of the DA system. A prospective study in non-human primates assessed the effects of 1-year treatment with MPH or placebo during adolescence. This positron emission tomography (PET) study found that the MPH group lacked the expected age-related decline observed in the placebo group, suggestive of increased D_2_/D_3_ receptors (Gill et al. 2012). In rats, juvenile treatment with MPH reduced DA transporter (DAT) densities immediately after treatment. When these rats were assessed in adulthood, DAT densities were even further reduced. In adult-treated rats, no such effect was observed (Moll et al. 2001). Another prospective study in rats showed that early MPH treatment persistently increased MPH-induced change in cerebral blood volume (CBV) in the thalamus, cingulate and medial prefrontal cortex (mPFC) later in life (Andersen et al. 2008). Behaviorally, juvenile MPH exposure reduced cocaine self-administration (Andersen et al. 2002), but increased anxiety and depressive-like behaviors in rats (Bolaños et al. 2003; Carlezon et al. 2003).

Human prospective studies are limited to short-term effects (and treatment durations) for ethical reasons. Data from a recent randomized controlled trial (RCT) from our group were in line with the above mentioned preclinical work, showing that four months of MPH treatment increased cerebral blood flow (CBF) response to acute MPH, in children, but not in adults with ADHD using phMRI (Schrantee et al. 2016). However, to study the lasting effects of early stimulant treatment, retrospective cohort studies can provide important information. For example, studies have shown both positive (Biederman et al. 2009; Mannuzza et al. 2008; Wilens et al. 2003) and negative (Molina et al. 2009) associations between stimulant treatment in adolescence and occurrence of substance-use disorders (SUDs), as well as anxiety and depressive disorders. However, the long-term effects of age-of-first stimulant treatment on DA development have not yet been studied in this context.

Taken together, the available evidence suggests that the effects of stimulants on the DA system are dependent on age-of-first-treatment, possibly reflecting ‘neurochemical imprinting’ (Andersen and Navalta 2004). This theory also predicts that these effects are only fully expressed when the system reaches maturation (e.g., typically during adulthood). Using a cohort-study, we here studied the relation between age-of-first-stimulant-exposure and the DA system using pharmacological magnetic resonance imaging (phMRI). phMRI is a non-invasive imaging technique to indirectly assess DA function, which indirectly measures DA neurotransmitter function by assessing hemodynamic changes induced by a dopaminergic drug challenge. These phMRI signal changes strongly correlate with DA release and DA transporter availability in preclinical and clinical studies (Chen et al. 1997; Schrantee et al. 2015).

We included three groups of adult ADHD patients: those that had either been exposed to stimulants early in life (before the age of 16), later in life (after the age of 23), or were naive to stimulant treatment. Based on the literature, we hypothesized a higher CBF response to an MPH challenge in early exposed individuals compared to late exposed, or stimulant treatment-naive individuals; higher anxiety and depression scores in early but not late exposed, or stimulant treatment-naive individuals; but less use of recreational drugs use in early, but not late- or stimulant treatment-naive individuals.

## Methods and materials

### Participants

Eighty-one male ADHD patients (23–40 years) were recruited via outpatient clinics, newspaper advertisements, databases containing prescription data (Pharmo Institute Utrecht) and the ePOD-MPH RCT (Schrantee et al. 2016). All subjects had a clinical diagnosis of ADHD requiring pharmacological treatment with a stimulant (diagnosed by psychiatrist, psychologist or pediatrian (primarily) or GP according to DSM-III or DSM-IV criteria according to Dutch treatment guidelines). Exclusion criteria were: IQ < 80, history of brain trauma or neurological disease, MRI contra-indications and substance use (including cocaine, heroin, synthetic drugs, or alcohol) meeting diagnostic criteria for abuse/dependence. Subjects were stratified into three exposure groups: 1) *early stimulant treatment (EST)* group: subjects treated with stimulants for at least four months before the age of 16 years 2) a *late stimulant treatment (LST)* group: subjects treated with stimulants for at least four months after the age of 23 years and 3) a *stimulant treatment-naive (STN)* group: containing subjects with no history of stimulant medication. Four months of treatment was chosen in line with effects found on CBF in MPH-treated children in a prospective study (Schrantee et al. 2016). The age limit of the EST group was chosen because this coincides with the end of puberty in boys, and because preclinical studies have reported effects of treatment in early adolescence (Bottelier et al. 2014). Self-reported prescription history were verified with available prescription data from pharmacies and treating physicians. The study was carried out in accordance with the Declaration of Helsinki (2012) and was approved by the Medical Ethical Committee. All subjects gave written informed consent.

### Procedures

Subjects underwent two phMRI scan sessions, in which we assessed the CBF response to an acute challenge to MPH, as a proxy for DA functionality (Chen et al. 1997; Schrantee and Reneman 2014). The first phMRI scan session was immediately followed by an oral challenge of MPH of 0.5 mg/kg MPH (with a maximum dose of 40 mg). The second scan session was conducted after 90 min, which is the time after which peak plasma levels of MPH are reached (Swanson and Volkow 2003). In both sessions an arterial spin labeling (ASL) scan was obtained to assess CBF in the fronto-striatal circuitry. All subjects were medication-free for at least a week before the scan, to prevent acute effects of stimulant treatment on CBF. In addition, subjects were instructed to abstain from drugs of abuse at least one week before the study, alcohol at least 24 h before the study and not to use caffeine or tobacco on the study day.

### MRI acquisition and image analysis

Data were acquired using a 3.0 T Philips MR Scanner (Philips Medical Systems, Best, The Netherlands). First, an anatomical 3D–FFE T1-weighted scan was obtained with the following scan parameters: TR/TE = 9.8/4.6; FOV = 256x256x120; voxel size = 0.875 × 0.875 × 1.2 mm. CBF images were acquired using a pseudo continuous arterial spin labeling (pCASL) sequence with the following parameters: TR/TE = 4000/14 ms; post-labeling delay = 1650 ms; label duration = 1525 ms; FOV = 240x240x119; 75 dynamics; voxel size = 3x3x7mm, GE-EPI, SENSE = 2.5, no background suppression, scan time = 10 min. Heart rate (HR) was monitored using a peripheral pulse unit.

**Fig. 1:**
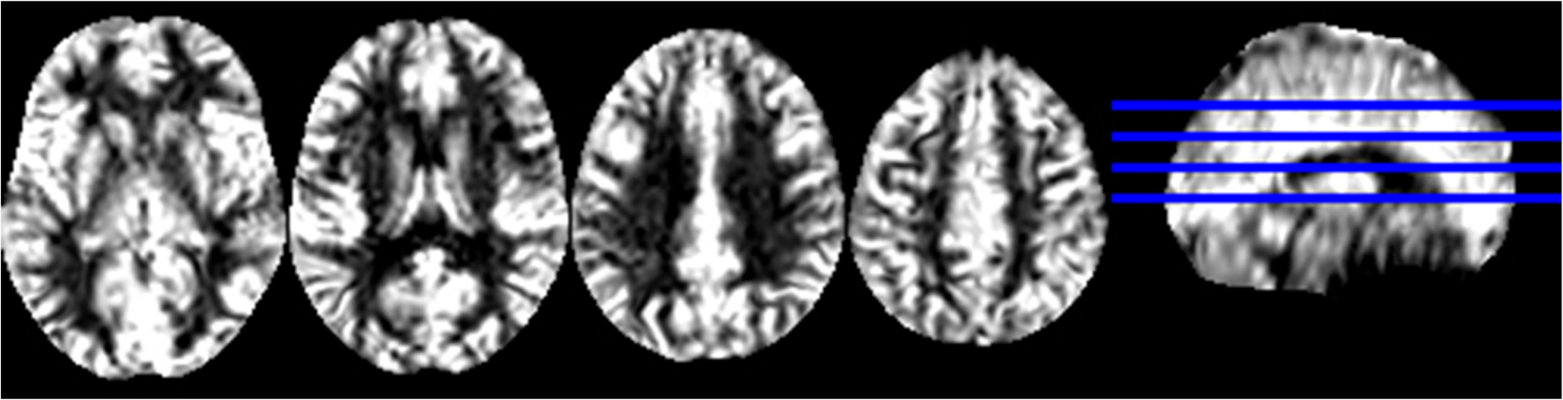
Axial view of a perfusion-weighted ASL scan from a representative subject

Data were processed using the Iris pipeline for CBF quantification and multi-atlas region segmentation (Bron et al. 2014). All image registrations were performed using Elastix registration software (Klein et al. 2010). For the ASL data, motion estimation was performed using rigid registration with a group-wise method that uses a similarity metric based on principal component analysis. Then, outlier rejection was performed to correct for sudden head movements. Outlier rejection was based on the M_diff_ images, the subtractions of all pairs of control (M_c_) and label images (M_l_). For each pair of M_diff_ images, we computed the sum of squared differences (SSD) which is the sum of all squared voxel-wise differences between the two images. As such, for each of the 75 time points, we obtained 74 SSD values over which we computed the median and SD. To obtain a more robust estimate of the SD, we computed this based on only the SSD values that were lower than the median. If more than 50% of the SSD values were larger than the median + (3*SD) this timepoint was considered an outlier. Subsequently, motion correction was performed on the remaining timepoints, and the resulting motion-compensated M_diff_ images were averaged to obtain a perfusion-weighted image (ΔM). Motion was quantified as the mean framewise displacement. Quantification of CBF was performed using the single-compartment model (Buxton et al. 1998), which is the recommended approach for pCASL (Alsop et al. 2014). The following parameters were used: labeling efficiency αGM = 0.85, T1GM = 1.6 ms, blood-brain partition coefficient λGM = 0.95 mL/g. The average of M_c_ images was used as a proton-density normalization image (M_0_) for the CBF quantification. Differences in post-labeling delays between slices (due to the 2D read-out) were accounted for. CBF was quantified in GM only, with a 3D method for partial volume correction based on local linear regression using the tissue probability maps (Asllani et al. 2008; Oliver et al. 2012). For each subject, probabilistic GM segmentations based on the T1-weighted scan (SPM8, Statistical Parametric Mapping, UCL, London, UK) were rigidly registered to the ΔM images by maximizing mutual information. For further analysis, CBF maps were transformed to the space of the T1-weighted scan. An example of a representative perfusion-weighted image can be observed in Fig. 1.

**Fig. 2:**
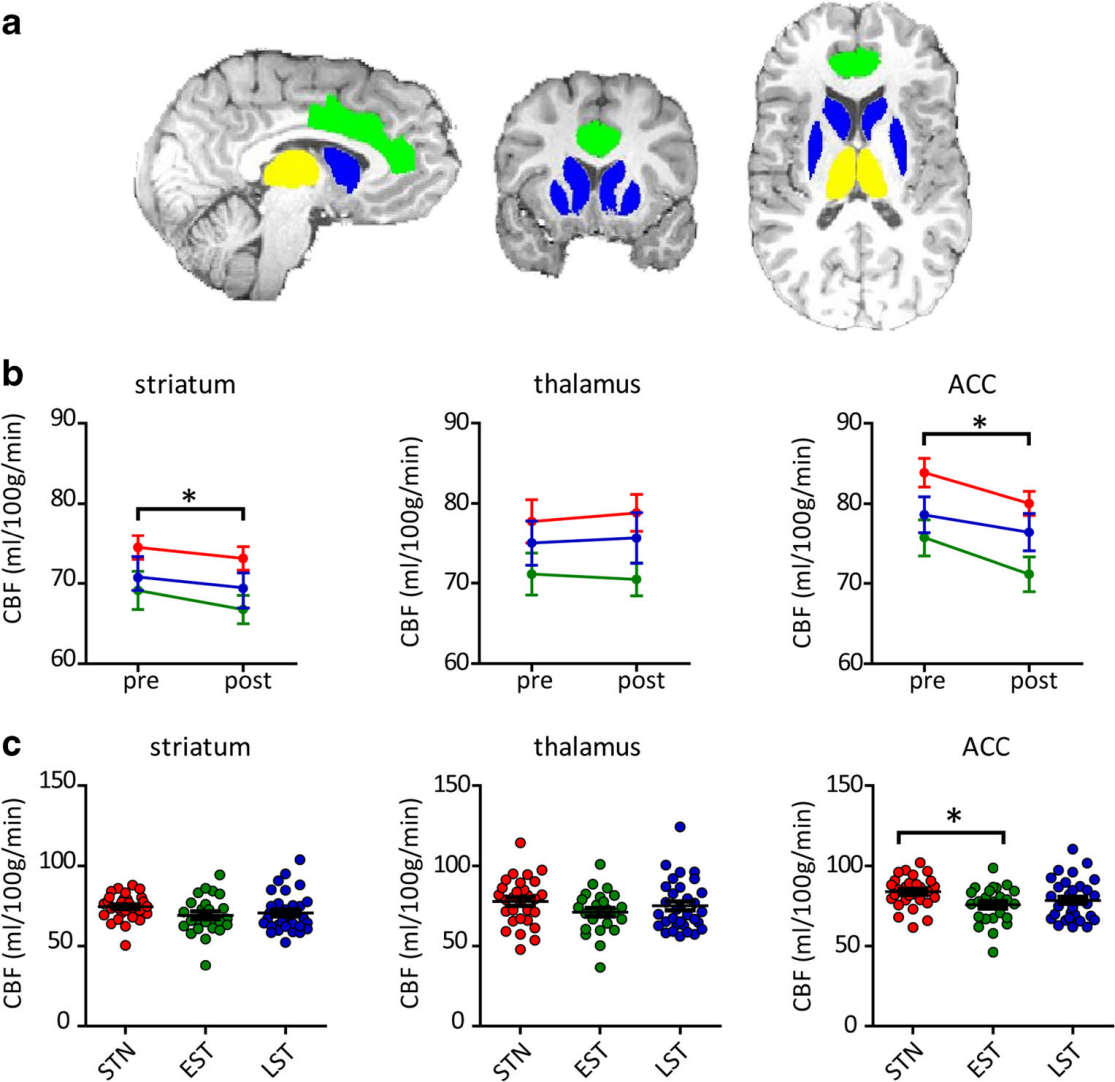
**a** Regions of interest used for analyses. *Blue* = striatum ; *green* = anterior cingulate cortex; *yellow* = thalamus. **b** change in CBF (ml/100 g/min) following acute MPH challenge (oral, 0.5 mg/kg) in the striatum, thalamus and anterior cingulate cortex (ACC). There was a main effect of challenge in the striatum and ACC, but not the thalamus. We found no group*time interaction in any of the ROIs. Mean and standard error of the mean are displayed. **c** scatter dot plot of CBF baseline values (ml/100 g/min) for all subjects. The *EST* group demonstrated significantly lower CBF than the *STN* group in the ACC only *p < 0.05. *red* = STN; *green* = EST; *blue* = LST

For each participant, we defined three regions of interest (ROIs) using a multi-atlas approach, registering 30 labeled T1-weighted images (Gousias et al. 2008; Hammers et al. 2003) with the participants’ T1-weighted images. The labels of the 30 atlas images were fused using a majority voting algorithm to obtain a final ROI labeling (Heckemann et al. 2006). For three pre-defined ROIs, comprising the striatum, thalamus and anterior cingulate cortex (ACC), CBF mean values were extracted (Fig. 2). The striatum was selected because it is rich in DAT (the primary target of action of MPH) and the thalamus and prefrontal cortex were chosen because the animal literature demonstrated largest effects of early MPH treatment using phMRI in these two important neuronal projections from the striatum (Andersen et al. 2008).

### Rating scales and questionnaires

Premorbid intellectual function was estimated using the National Adult Reading test (Dutch version). Current ADHD symptom severity was assessed using the ADHD-Rating Scale (ADHD-RS) (Kooij et al. 2008). Current mood and anxiety symptoms were evaluated using the Beck Depression Inventory (BDI) and Beck Anxiety Inventory (BAI), respectively. In addition, lifetime recreational drug use was assessed using a drug history questionnaire.

### Statistical analyses

SPSS version 22.0 (IBM, Armonk, NY, USA) was used for all statistical testing. Data were assessed for normality. To assess the effect of age-of-first-exposure on CBF response to MPH we used a repeated-measures analysis of variance (ANOVA) for each ROI separately with group (*EST, LST* or *STN*) as a between-subjects factor and MPH-challenge (pre- or post-MPH) as a within-subjects factor, with subsequent post-hoc Sidak’s tests. Baseline differences in CBF were assessed using an univariate ANOVA. To further examine this age-dependency, we correlated age-of-first-exposure with CBF response and mood symptoms in the *LST* and *EST* group. In additional exploratory analyses, we assessed the effect of treatment duration and time-since-last-use. Differences in recreational drug use were assessed for cannabis, 3,4-methylenedioxy-methamphetamine (MDMA), cocaine and amphetamine using a χ^2^ test. To this end, subjects were divided in users (for cannabis >1× per week, for other drugs >10× lifetime) and non-users.

## Results

### Patient characteristics

Age and estimated IQ differed statistically significantly between the three groups of ADHD subjects, with the *EST* group being slightly younger and having a lower IQ than the *STN* and *LST* group (Table 1). In addition, current symptom severity was significantly higher in the *STN* group compared to the *EST* and *LST* group. Inherent to the design of the study, the *EST* group started medication treatment at a younger age and was treated for a much longer period of time (94.9 vs 11.8 months) than the *LST* group.

**Table 1:** Participant characteristics

	EST			LST			STN		
	*N* = 26			*N* = 29			*N* = 26		
	Mean	SD	Range	Mean	SD	Range	Mean	SD	Range
Age (years)	26.0	2.8	23–35	28.5	4.9	23–40	29.0	4.7	23–39*
Estimated IQ	100.3	8.0	82–113	108.0	8.6	92–124	107.7	6.4	95–118*
	Median	SD	Range	Median	SD	Range	Median	SD	Range
Age first stimulant treatment (years)	9.5	3.0	4–14	26	4.5	23–39	NA	NA	NA*
Treatment duration (months)	72	56.1	18–228	4	23.3	4–120	NA	NA	NA*
Time since last treatment (months)	84	57.2	0–168	0	1.3	0–6	NA	NA	NA*
ADHD-SR	23.2	10.0	1–45	21.8	7.6	10–39	30.4	10.3	8–50*
BDI	10.7	8.9	0–26	5.3	4.1	0–14	8.0	6.1	0–20*
BAI	8.5	8.7	0–35	6.3	4.9	0–18	9.4	7.7	0–25
Drug use									
Cannabis (% of subjects > cutoff)^a^	25%			55%			60%		*
MDMA (% of subjects > cutoff)^b^	13%			24%			31%		
Cocaine (% of subjects > cutoff)^b^	0%			24%			19%		*
Amphetamine (% of subjects > cutoff)^b^	0%			7%			8%		

*p < 0.05

^a^ more than once a week

^b^ more than 10 x lifetime

### Baseline CBF and MPH-induced changes in CBF

One patient did not complete the second ASL scan and was removed from the analysis. Motion during the MRI scan did not differ between the three groups (baseline: F(2,78) = 1.13, *p* = 0.33; change: F(2,78) = 0.75 *p* = 0.48). The MPH challenge increased HR (*p* < 0.01), but this effect did not differ between the three groups (F(2,75) = 1.51 *p* = 0.23). ANOVA revealed a significant effect of group on baseline CBF in the ACC (F(2,78) = 3.62, *p* = 0.03), but not in the striatum (F(2,78) = 2.07, *p* = 0.13) or thalamus (F(2,78) = 1.51, *p* = 0.23). Post-hoc tests showed that the *STN* group had a higher ACC CBF than the *EST* group (*p* = 0.03), but not compared to the *LST* group (*p* = 0.36) (Fig. 2). The acute MPH challenge reduced CBF (ΔCBF) in the striatum (F(80,1) = 6.69, *p* = 0.01) and ACC (F(80,1) = 20.28, *p* < 0.01), but not the thalamus (F(80,1) = 0.12, *p* = 0.73) (Fig. 2). However, no significant interaction effects were observed between group and ΔCBF in the ROIs studied, nor did we find a significant correlation between ΔCBF and age-of-first-exposure (*r* < 0.2 for all ROIs), treatment duration (*r* < 0.1 for all ROIs) or time-since-last-treatment (*r* < 0.1 for all ROIs). None of the results were affected by adding age, ADHD symptom severity or baseline CBF values as covariates to the model.

### Depression, anxiety and recreational drug use

We found a significant overall effect of group on depressive symptoms, (F(75,2) = 4.57, *p* = 0.01), but not on symptoms of anxiety (F(76,2) = 1.38, *p* = 0.26). Post hoc analyses revealed higher BDI scores in the *EST* than the *LST* group (*p* < 0.01). The *EST* individuals indicated using less cannabis, MDMA, cocaine as well as amphetamine than the *LST* and *STN* individuals, although this was only statistically significant for cannabis and cocaine (Table 1).

## Discussion

Here we investigated if age of first stimulant exposure modulates the effect CBF response to MPH, mood and anxiety symptoms as well as recreational drug use. We did not find a different CBF response to MPH between groups, but the *EST* group showed lower baseline ACC CBF than the *STN* group, which could be a result of early-induced changes by stimulants to the developing DA system. In line with this, and as hypothesized, the EST group showed higher depression- but not anxiety-levels and reported less recreational drug use.

### Long-term effects of stimulants on CBF

#### Modulation by age of stimulant exposure: baseline

To our knowledge, this is the first study investigating the long-term effects of age-of-first-exposure on the DA system in humans. The DA system is in development all throughout childhood and adolescence. For example, cortical D_2_/D_3_ expression peaks in early childhood, followed by a sharp decline during adolescence (Seeman et al. 1987), whereas dopamine transporter (DAT) density peaks mid-adolescence while slowly declining thereafter (Meng et al. 1999). In non-human primates MPH treatment during adolescence resulted in less decline of striatal D_2_/D_3_ receptor binding following one year of MPH treatment compared to the placebo group, suggesting halted development of these receptors (Gill et al. 2012). In line with that study, lower CBF in the ACC in the *EST* group compared to the *STN* group, as we observed here, might reflect higher density of D_2_/D_3_ receptors induced by early treatment, because experimental phMRI studies in rats have shown that negative rCBV responses reflect agonism of D_2_/D_3_ receptors, whereas positive rCBV changes are associated with agonism of D_1_/D_5_ receptors (Chen et al. 2010). However, this interpretation is speculative; Gill et al. (2012) found changes in the striatum, whereas we reported changes in the ACC. Future studies will need to study the changes in DA function and connectivity within all areas of the fronto-thalamo-striatal loops in more detail. Moreover, we did not find significant differences in ACC CBF between the *EST* and *LST* group and therefore caution is needed in interpreting the age-dependency of this effect.

In accordance with predictions from the neuronal imprinting theory, we did not find differences in baseline CBF between the *LST* and *STN* group. This is in contrast with a study in adult ADHD patients, showing increased DAT following one year of stimulant medication (Wang et al. 2013). Increased DAT availability could result in lower CBF because of less availability of extracellular DA, because less DA release results in relatively more D_2_/D_3_ receptor stimulation. However, they measured DAT 24 h after the last clinical dose of MPH (Wang et al. 2013), whereas we conducted our phMRI scans at least one week after treatment cessation. Although 24 h should ensure dissipation of acute MPH effects, transient up-regulation of DAT cannot be excluded in that study.

#### Modulation by age of stimulant exposure: MPH challenge

Our findings of reductions in CBF in the fronto-striatal circuitry after an acute challenge with MPH are in agreement with studies comparing on/off medication periods in adult ADHD patients (O’Gorman et al. 2008; Schweitzer et al. 2003). Studies in healthy volunteers report more mixed results. In adult volunteers, MPH induced increased CBF in striatum and thalamus in an ASL study (Marquand et al. 2012), but increased CBF in the anterior cingulate, supplementary motor areas and temporal poles in a H_2_[O^15^] PET study (Udo de Haes et al. 2007). Decreased CBF was reported in lateral frontal, rostral cingulate and sensorimotor areas, amygdala, parahippocampal gyrus and in multiple regions of the occipital and temporal cortices for the ASL study, but in superior temporal gyri, right medial frontal gyrus, and right inferior parietal cortex for the PET study. One reason for the discrepancy between studies in volunteers and ADHD patients might be altered DA release in ADHD patients (Cherkasova et al. 2014; Volkow et al. 2007).

We found that in *EST* individuals with a mean treatment duration of eight years, CBF response to MPH was similar to that of *LST* and *STN* subjects. This finding was in contrast to our hypothesis, as preclinical studies have suggested that juvenile administration will result in DA changes that will last and possibly even expand as the brain matures (Moll et al. 2001). This hypothesis was supported by our RCT showing that four months of MPH treatment induced increased striatal and thalamic CBF response to a MPH challenge in stimulant-treatment naive children, but not adults with ADHD (Schrantee et al. 2016). The current results suggest that at least a part of this effect on the developing DA system is transient or compensated. Interestingly, we did not find a difference in CBF response to MPH between LST and STN subjects, suggesting an absence of tolerance to MPH following long-term treatment in adulthood. Interestingly, Volkow et al. (2012), found reduced striatal, but also no increased extrastriatal DA release to a DA challenge after 12-month MPH treatment in adults ADHD patients. Also in recreational dexamphetamine (dAMPH) users we observed a blunted striatal CBF response to dAMPH (Schrantee et al. 2015). However, recreational use of stimulants is usually associated with high dose binges, whereas much lower doses are used for stimulant treatment of ADHD.

### Long-term modulating effect of age of stimulant exposure on behavior

Although the short-term benefits of stimulants on ADHD symptoms are well-established, studies on long-term efficacy are inconclusive (van de Loo-Neus et al. 2011). Here, we observed lower ADHD symptom severity in the *EST* and *LST* group compared to the *STN* group, whereas the stimulant-treated groups did not differ, despite the long time since last exposure in the *EST* group. Our findings not only suggest that MPH is useful in reducing symptoms in adult ADHD (the *LST* group), but also suggests that the effects of treatment in the *EST* group are long-lasting.

Animal studies have suggested an increased risk for depressive symptoms following MPH exposure early in life (Bolaños et al. 2008; Carlezon et al. 2003), but results from human studies are equivocal (Biederman et al. 2009; Mannuzza et al. 2008; Molina et al. 2009; Wilens et al. 2003). One limitation of our study is that, as a result of the study design, children with both ADHD and depressive symptoms could have been more likely to receive treatment at young age and thus end up in our *EST* group. In the current study, we observed that *EST* subjects have more depressive symptoms (~mild-moderate depression) than the other groups. This is in line with a transient increased anxiety and depression in the MTA trial (Molina et al. 2009), but in contrast with studies reporting protective effects of stimulant use on symptoms of anxiety and depression (Biederman et al. 2009; Daviss et al. 2008; Lee et al. 2016).

In the current study we could not assess the effect of treatment on SUDs, as this was an exclusion criterion; however a large number of subjects in this sample were recreational drug users. Interestingly, we found lower drug use in the *EST* group compared to the *STN* and *LST* group, especially regarding MDMA and cocaine use. This is line with literature showing that whereas adult ADHD is associated with a high rate of substance abuse (Dalsgaard et al. 2014), childhood treatment does not increase (Humphreys et al. 2013; Molina et al. 2013), and may even decrease this risk (Spencer et al. 2006). Our findings are also consistent with the literature on self-medication in ADHD (Wilens 2004), suggesting that ADHD patients not taking stimulants are more likely to use drugs to alleviate behavioral symptoms. An alternative explanation, and not necessarily incompatible, is that lower vulnerability for SUDs may be due to changes in the dopaminergic reward system following early stimulant treatment.

### Methodological considerations

The cohort we studied was heterogeneous in terms of symptom onset, treatment duration, symptom severity and probably also the course of the disorder. Furthermore, the interaction between development, age-of-first stimulant treatment, and duration of treatment is likely not linear, which needs to be taken into account for future studies. As a long-term RCT would not be ethical, we have to rely on pre-clinical studies and retrospective cross-sectional studies to inform us about possible long-term effects of stimulants on the DA system. Currently, many imaging initiatives are established to share clinical and imaging data, which could facilitate replication of small hypothesis-driven studies, such as this one, in larger samples.

ASL-phMRI is an indirect method to measure neurotransmitter function. Previous studies have shown that phMRI closely parallels DA function (Chen et al. 1997). Nevertheless, as we measure a vascular response to neuronal function, it is possible that the CBF changes are caused by alterations in other neurotransmitter systems, such as the noradrenalin system, or mediated in part by cardiovascular effects. However, even though HR increased following MPH, we did not find differences between the groups and therefore cardiovascular effects are unlikely to explain our results. We used a fixed-order, open-label design for two reasons; first, CBF varies considerably across days and therefore a baseline scan followed by an MPH scan was preferred. Second, participants can easily discriminate between MPH and placebo and therefore blinding was not possible in this study. Poly-drug use is a limitation in this study and because of the high association between ADHD and drug (ab)use it is difficult to correct for or quantify the possible effect on our results. In addition, we cannot exclude that the increased depressive symptoms in the *EST* group are a pre-existing vulnerability, instead of consequence of early stimulant treatment.

### Conclusion

Our results suggest long-lasting effects of early stimulant treatment on baseline CBF, ADHD symptoms, mood as well as recreational drug use. Nevertheless, we did not find lasting effects of stimulant exposure on the phMRI response, suggesting that at least some effects on the developing DA system are transient or compensated for. It is likely that the neurochemical imprinting effect of stimulant treatment on the DA system is a dynamic process. Our data thus stress the need for prospective follow-up studies including assessment at multiple ages to completely characterize the long-term effects of ADHD medication on the human brain.
